# Fanconi-BRCA pathway mutations in childhood T-cell acute lymphoblastic leukemia

**DOI:** 10.1371/journal.pone.0221288

**Published:** 2019-11-13

**Authors:** Gayle P. Pouliot, James Degar, Laura Hinze, Bose Kochupurakkal, Chau D. Vo, Melissa A. Burns, Lisa Moreau, Chirag Ganesa, Justine Roderick, Sofie Peirs, Bjorn Menten, Mignon L. Loh, Stephen P. Hunger, Lewis B. Silverman, Marian H. Harris, Kristen E. Stevenson, David M. Weinstock, Andrew P. Weng, Pieter Van Vlierberghe, Alan D. D’Andrea, Alejandro Gutierrez

**Affiliations:** 1 Division of Hematology/Oncology, Boston Children’s Hospital, Boston, Massachusetts, United States of America; 2 Department of Pediatric Oncology, Dana-Farber Cancer Institute, Boston, Massachusetts, United States of America; 3 Center for DNA Damage and Repair and Department of Radiation Oncology, Dana-Farber Cancer Institute, Boston, Massachusetts, United States of America; 4 Department of Molecular, Cell and Cancer Biology, University of Massachusetts Medical School, Worcester, Massachusetts, United States of America; 5 Department of Biomolecular Medicine, Ghent University, Ghent, Belgium; 6 Department of Pediatrics, University of California San Francisco, San Francisco, California, United States of America; 7 Division of Oncology and the Center for Childhood Cancer Research, The Children’s Hospital of Philadelphia, Philadelphia, Pennsylvania, United States of America; 8 Department of Pathology, Boston Children’s Hospital, Boston, Massachusetts, United States of America; 9 Department of Biostatistics and Computational Biology, Dana-Farber Cancer Institute, Boston, Massachusetts, United States of America; 10 Department of Medical Oncology, Dana-Farber Cancer Institute, Boston, Massachusetts, United States of America; 11 Terry Fox Laboratory, British Columbia Cancer Agency, Vancouver, British Columbia, Canada; Ohio State University, UNITED STATES

## Abstract

BRCA2 (also known as FANCD1) is a core component of the Fanconi pathway and suppresses transformation of immature T-cells in mice. However, the contribution of Fanconi-BRCA pathway deficiency to human T-cell acute lymphoblastic leukemia (T-ALL) remains undefined. We identified point mutations in 9 (23%) of 40 human T-ALL cases analyzed, with variant allele fractions consistent with heterozygous mutations early in tumor evolution. Two of these mutations were present in remission bone marrow specimens, suggesting germline alterations. BRCA2 was the most commonly mutated gene. The identified Fanconi-BRCA mutations encode hypomorphic or null alleles, as evidenced by their inability to fully rescue Fanconi-deficient cells from chromosome breakage, cytotoxicity and/or G2/M arrest upon treatment with DNA cross-linking agents. Disabling the tumor suppressor activity of the Fanconi-BRCA pathway is generally thought to require biallelic gene mutations. However, all mutations identified were monoallelic, and most cases appeared to retain expression of the wild-type allele. Using isogenic T-ALL cells, we found that *BRCA2* haploinsufficiency induces selective hypersensitivity to ATR inhibition, in vitro and in vivo. These findings implicate Fanconi-BRCA pathway haploinsufficiency in the molecular pathogenesis of T-ALL, and provide a therapeutic rationale for inhibition of ATR or other druggable effectors of homologous recombination.

## Introduction

The Fanconi pathway functions in the repair of DNA inter-strand crosslinks and stalled replication forks [1]. Stalled replication forks trigger activation of the upstream Fanconi complex (or Fanconi complex 1), which ubiquitinates FANCD2. FANCD2 ubiquitination promotes the formation of the downstream Fanconi complex (complex 2), which mediates subsequent DNA repair. Biallelic germline mutations of any Fanconi genes including *BRCA2* cause Fanconi anemia, a syndrome characterized by developmental anomalies, bone marrow failure, and a predisposition to acute myeloid leukemia and squamous cell carcinomas [1].

The study of rare genetic cancer predisposition syndromes has revealed fundamental insights into the pathobiology of sporadic oncogenesis in the general population. For example, familial adenomatous polyposis, a clinical syndrome associated with highly penetrant colorectal adenocarcinomas [2], is caused by germline mutations of APC [3–5]. Although familial adenomatous polyposis accounts for only a small fraction of colorectal cancers in the general population, APC gene mutations are the most common genetic alteration in sporadic colorectal adenocarcinomas [3, 6]. Likewise, germline monoallelic mutations of *BRCA1* and *BRCA2* cause familial breast and ovarian cancer predisposition syndromes [7, 8], and these genes are recurrently mutated in sporadic breast and ovarian cancers [9, 10]. While germline or somatic mutations of these and other Fanconi genes, including upstream Fanconi genes such as *FANCM*, have been linked to several tumor types, these are most often carcinomas [9–15]. Given that the pathobiology of sporadic cancers is shared with that of genetic cancer predisposition syndromes across numerous tumor types, the paucity of Fanconi-BRCA mutations reported in sporadic human leukemias would appear to be discordant with the high incidence of leukemia in patients with Fanconi anemia [16].

*Brca2-*deficient mice spontaneously develop T-lymphoblastic malignancies at high frequency [17, 18], and these tumors also occur in *Fancc*-deficient mice [19]. Furthermore, several case reports have described T-lymphoblastic malignancies in individuals with germline mutations of the Fanconi-BRCA pathway [20–24]. However, the degree to which this pathway plays a role in human T-cell leukemogenesis remains unclear. Here, we report a high frequency of loss-of-function mutations of Fanconi-BRCA pathway genes in childhood T-ALL. All mutations identified were monoallelic, and most of these cases retained expression of the wild-type allele, suggesting partial rather than complete Fanconi-BRCA pathway inactivation. Despite the clinical benefit of PARP inhibitors in tumors with complete Fanconi-BRCA pathway inactivation [25, 26], this approach appears to have little efficacy against tumors with monoallelic BRCA gene mutations [27]. However, we found that *BRCA2* haploinsufficient cells are hypersensitive to ATR inhibition, in vitro and in vivo. Thus, BRCA haploinsufficiency may represent a targetable pathogenic alteration in T-ALL.

## Materials and methods

### Patient samples

The primary cohort of T-ALL diagnostic samples consisted of 40 specimens collected from children with newly diagnosed T-ALL who were enrolled on Dana-Farber Cancer Institute Study 05–001 between April 2005 and February 2010 (https://clinicaltrials.gov/ct2/show/NCT00400946), or Children’s Oncology Group Study AALL0434 between January 2007 and June 2014 (https://clinicaltrials.gov/ct2/show/NCT00408005). The validation cohort consistent of a distinct 69 pediatric T-ALL patient samples collected from children on Dana-Farber Cancer Institute Study 05–001 over the same time period. All samples were obtained prior to the initiation of chemotherapy. Samples were purified using Ficoll-Paque reagent, and cells were viably frozen. All samples were collected with written informed consent and Institutional Review Board (IRB) approval of the respective institutions, in accordance with the Declaration of Helsinki.

### Targeted exon sequencing of T-ALL patient samples

Targeted exon sequencing for all protein-coding exons of the genes listed in S1 Table was performed using the customized Dana-Farber OncoPanel massively parallel sequencing assay as described [28]. Briefly, DNA was isolated using the AllPrep DNA/RNA Mini Kit (OPv1 cohort, Qiagen, Venlo, The Netherlands) or the DNeasy kit (OPv3mod cohort; Qiagen) according to manufracturer’s instructions and DNA concentration analyzed using PicoGreen. DNA library preparation and hybrid capture were performed as described [28], and samples were sequenced on an Illumina HISeq 2500. Pooled sample reads were de-convoluted and sorted using Picard tools (https://github.com/broadinstitute/picard), and aligned to the human genome using build 37 edition from the Human Genome Reference Consortium, as described [28]. Mutation analysis for single nucleotide variants (SNV sheet in S3 Table) was performed using MuTect v1.1.4. Analysis for insertions/deletions (INDEL sheet in S3 Table) was performed using the SomaticIndelDetector tool. Analysis for multiple CNVs in the same codon was performed using the GATK ReadBackPhasing Tool (PHASED sheet in S3 Table), as described [29]. The Best Effect single nucleotide variant (SNV) and indel data were then filtered using the 6,500 exome release of the Exome Sequencing Project (ESP) database and queried against dbSNP and COSMIC database. Fanconi-BRCA pathway genes sequenced included FANCA, FANCC, FANCE, FANCF and FANCG, BRCA2/FANCD1, FANCD2, BRIP1/FANCJ, PALB2/FANCN, and ERCC4/FANCQ. BRCA1, which has recently been shown to also be a Fanconi gene [30], was also sequenced.

Mutation calls were made for mutations with an allele fraction ≥ 0.3, and the mutation was predicted to result in a non-synonymous amino acid alteration, or predicted to disrupt a start codon, stop codon, or splice site. Mutations were excluded if they were present at any frequency in dbSNP or in the Exome Variant Server, NHLBI GO Exome Sequencing (http://evs.gs.washington.edu/EVS/) at the time of analysis. Data from targeted exon sequencing and RNA sequencing of primary T-ALL patient samples is available in the dbGap controlled-access database (https://www.ncbi.nlm.nih.gov/gap), study ID: phs001513, which is accessible to users with the appropriate institutional certifications for human subject projections.

### Array CGH analysis of T-ALL patient samples

All samples analyzed by targeted exon sequencing in the primary cohort that had sufficient material available (n = 36 of 40 in OPv1 cohort) were also profiled for DNA copy number analysis using SurePrint G3 Human 4×180K CGH Microarrays (Agilent Technologies, Santa Clara, CA). Patient and control genomic DNAs (gDNAs) were labeled with Cy3 and Cy5 dyes (PerkinElmer, Waltham, MA) and hybridization was performed according to the manufacturer’s instructions (Agilent Technologies), followed by data-analysis using the arrayCGHbase tool [31]. Segmentation was performed with the BioConductor DNAcopy package (http://www.bioconductor.org/packages/2.2/bioc/html/DNAcopy.html), as previously described [32]. Log_2_ copy number ratio for heterozygous deletion was defined as -0.5 to -1.5 (corresponding to 35–70% of normal copy number), and log_2_ copy number ratio for homozygous deletion was defined as less than -1.5 (corresponding to <35% of normal copy number). Log_2_ copy number ratio for duplication was 0.32 to 0.81 (corresponding to 25 to 75% of normal copy number), and Log2 copy number ratios greater than 0.81 were considered amplification. Array CGH data are available in the NCBI Gene Expression Omnibus (https://www.ncbi.nlm.nih.gov/geo/) as GSE96624.

### Sanger sequencing

For Sanger sequencing of genomic DNA, PCR amplification of the region of interest surrounding each mutation was performed using the OneTaq Hot Start kit (Qiagen, Hilden, Germany) with the forward (F) and reverse (R) primers listed in S6 Table. PCR products were then purified using the QIAquick PCR purification kit (Qiagen) and sent for sequencing with the sequencing primers listed at S6 Table.

For sequencing of the cDNA, RNA was isolated from the samples using the AllPrep Kit (Qiagen) as above, treated with DNase I (Thermo Fisher, Waltham, MA), and then used for cDNA synthesis using the SuperScript III First-Strand Synthesis System (Thermo Fisher). The cDNA product was then used for PCR amplification with the primers listed in S6 Table using the OneTaq Hot Start kit (Qiagen). The PCR product was purified using QIAquick PCR purification kit (Qiagen) and sent for sequencing. Sanger sequencing was performed by Genewiz (South Plainfield, NJ).

### Patient derived xenografts

Viable T-ALL blasts collected from children with T-ALL were engrafted and expanded in immunodeficient mice, as described [33]. Cells were cultured in alpha-minimum essential media supplemented with 10% FBS, 10% human AB serum (Invitrogen), 1% L-glutamine, 1% penicillin/streptomycin in the presence of recombinant human cytokines stem cell factor (50 ng/ml), Flt3-L (20 ng/ml) and interleukin-7 (10 ng/ml; R&D Systems, Minneapolis, MN, USA) at 37 °C under 5% CO_2_. All mouse experiments were approved by the Boston Children’s Hospital Institutional Animal Care and Use Committee, and performed in accordance with all regulations.

### Fanconi deficient cell lines

The following Fanconi deficient cell lines were used: VU423 (also known as EUFA423) cells, which harbor compound heterozygous *BRCA2* inactivating mutations [34]; PD20 cells, which harbor compound heterozygous *FANCD2* inactivating mutations [35]; GM6914 cells, which are *FANCA* deficient [36]; PD331 cells, which are *FANCC* deficient [37]; EUFA121.L cells, which have compound heterozygous inactivating mutations of *FANCF* [38]; and HSC 563.L cells, which are deficient for FANCE. All cell lines were obtained from the Alan D’Andrea laboratory or the Oregon Health Sciences University Fanconi Anemia Cell Repository (http://www.ohsu.edu/research/fanconi-anemia/celllines.cfm). EUFA 121.L cells were cultured in RPMI with 15% FBS with 1% penicillin and streptomycin (P/S). VU423, GM6914, PD20 and PD331 cells were cultured in DMEM with 10% FBS with 1% P/S.

### Fanconi expression constructs and site-directed mutagenesis

A pcDNA3 plasmid was used to drive expression of wild-type BRCA2 and G418 resistance. A PMMP retroviral vector was used to drive expression of FANCD2, FANCA, FANCC, or FANCF and puromycin resistance. Site-directed mutagenesis was performed on the wild-type plasmids to generate expression constructs encoding each of the T-ALL-associated mutations shown, using the QuikChange Lightning Site-Directed Mutagenesis Kit (Agilent Technologies, Santa Clara, CA) and the primers in S6 Table. All plasmids were Sanger sequenced to confirm correct introduction of the patient-derived mutations.

### Lentiviral and retroviral production and transduction of cell lines

Viral particles for PMMP-driven expression constructs for FANCD2, FANCA, FANCC, or FANCF were generated by co-transfecting these plasmids into HEK 293T cells together with packaging plasmids pMD and p-CMV-VSV-G using Fugene 6 (Promega, Madison, WI). Lentiviruses were generated by co-transfecting pLenti CMV Puro DEST plasmid with packaging vectors psPAX2 (https://www.addgene.org/12260/) and pMD2.G (https://www.addgene.org/12259/) using Fugene (Promega, Madison, WI). The appropriate Fanconi-deficient cell lines were infected with each of the wild-type and point mutant constructs for that gene by incubating cells for 24 hours in virus-containing supernatant in the presence of polybrene at 8 μg/mL. Controls were pcDNA3-Luciferase (control for BRCA2), or PMMP empty vector (FANCD2, FANCA, FANCC, FANCF). Selection was with G418 (BRCA2) or puromycin (FANCD2, FANCA, FANCC, FANCF) at 2 μg/ml beginning 48 hours after infection for at least 72 hours.

The *BRCA2* coding sequence is too large for standard viral packaging, so cells were transfected with pcDNA3 constructs encoding wild-type or mutant BRCA2, or Luciferase control, using Lipofectamine 2000 (Thermo Fisher Scientific). Selection for successfully transfected cells was with G418 treatment at 500 μg/ml beginning 48 hours after transfection and continuing for a minimum of 10 days.

### Quantitative Reverse Transcriptase PCR and primers

RNA was isolated from cells transduced with the indicated constructs using the RNeasy kit (Qiagen), and cDNA was synthesized using the SuperScript III First-Strand Synthesis System using the oligo(dT)_20_ primers provided (Thermo Fisher Scientific). Q-RT-PCR was performed using Power SYBR Green PCR Master Mix (Thermo Fisher Scientific) and a 7500 Real-Time PCR system (Applied Biosystems) using the primers for each target gene in listed in S6 Table. Beta-actin was the control, and relative gene expression was calculated using the ΔΔCt method.

### Western Blotting

Cells were lysed with 10 mM HEPES pH 8.0, 8 M Urea, 1% CHAPS, 150 mM NaCl, 1 mM EDTA, 10 mM glycine supplemented with protease cocktail inhibitor (Roche), resolved by NuPAGE (Invitrogen) gels (NuPAGE 4–12% Bis-Tris for FANCA, FANCC, FANCD2 and FANCF and NuPAGE Tris-Acetate 3–8% for BRCA2) and transferred onto nitrocellulose membrane. The following antibodies were used, FANCA (Bethyl Labs), FANCC (ID#3831, OHSU, Fanconi Anemia Antibody Project, Portland, OR, USA), BRCA2/FANCD1-1 (D0405, OHSU, Fanconi Anemia Antibody Project, Portland, OR, USA), FANCD2 (SC-20022, Santa Cruz, Dallas, TX, USA) FANCF (ab47624m, Abcam, Cambridge, MA, USA), beta-Actin (Cell Signaling Technologies, Danvers, MA, USA). Secondary antibodies were anti-mouse IgG, HRP-linked Antibody and anti-rabbit IgG, HRP-linked antibody (Cell Signaling Technologies, Danvers, MA, USA). Detection was with Supersignal^™^ West Pico PLUS chemiluminescent Substrate (Thermo Scientific, Waltham, MA,USA) using the Amersham Imager 600 (GE Healthcare Life Sciences).

### Mitomycin C complementation assays

Mitomycin C was obtained from Sigma-Aldrich (St. Louis, MO) and dissolved in water. For chromosomal breakage assays, cells were plated at 5 million cells/10 ml of media. Cells were treated with mitomycin C (20 ng/ml) or vehicle control (water) for 48 hours at 37°C with 5% CO_2_, at which time colcemid was added to final concentration of 100 ng/mL for 1 hour. Adherent cells were trypsinized and incubated in 10 ml hypotonic 0.075 M KCl for 20 minutes. Two ml of methanol:acetic acid (3:1) was added to each sample, samples were centrifuged at 500 g x 10 minutes, and supernatant was removed. Cells were then resuspended in 5 mL of methanol:acetic acid fix (3:1), centrifuged at 500 g x 10 minutes, a procedure that was repeated a total of 3 times after removing the supernatant. Cells were then dropped onto microscope slides and stained with Wright’s stain, after which 25 metaphase spreads from each of a minimum of 3 different slides was analyzed for each sample. Microscopy was performed using an 100x objective on an Axio Imager A1 microscope (Zeiss, Oberkochen, Germany), with images captured using a CV-A10 digital camera (Jai, Yokohama, Japan) and Cytovision software (Leica Biosystems, Wetzlar, Germany).

For cell viability complementation assays, cells were treated with mitomycin C at the indicated doses (between 50–500 nM) and incubated with drug for 96h following which cell viability was measured by CellTiter-Glo Luminescent Cell Viability Assay (Promega, Madison, WI).

### Cell cycle complementation assay

Cell cycle analysis was performed on cells treated with vehicle (DMSO) or melphalan at the indicated doses for 48h. Cells were then fixed with 70% ethanol. Following this, cells were washed with PBS and then stained with PI/RNase Staining Buffer (BD Biosciences, San Jose, CA, USA). Cell Cycle analysis was performed on the Beckton-Dickinson LSR Fortessa or BD LSR II (BD, Franklin Lakes, NJ, USA).

### Generation of isogenic *BRCA2* haploinsufficient vs. wild-type T-ALL cells

We used Jurkat cells, which lack an identifiable *BRCA2* mutation [39], to generate isogenic clones with monoallelic BRCA2 mutations. We first generated a guide RNA targeting exon 11 of *BRCA2* (NM_000059; guide RNA target sequence TACAACTTGTGTAAAAAGCTA) in the guide RNA expression vector pHKO9-combomod-Puro [40]. This guide RNA was packaged into an integration deficient lentivirus by transfection into 293T cells, together with the integration-defective packaging plasmid psPAX2-D64V (https://www.addgene.org/63586/) and the pMD2.G envelope plasmid (https://www.addgene.org/12259/).

Jurkat T-ALL cells were first transduced with a blasticin-resistant Cas9 expression lentivirus (https://www.addgene.org/52962/), and selected with blasticidin for 10 days. Cells were then transduced with the integration-defective *BRCA2*-targeting guide RNA by incubating cells for 24 hours in virus-containing supernatant in the presence of polybrene at 8 μg/mL, and then subjected to selection with puromycin (2 μg/ml) for 24 hours, followed by single cell cloning using serial dilution. The *BRCA2* target locus of these single-cell clones were Sanger sequenced using the following primers for PCR amplification: forward- 5-‘TGTGGTGCCACCTAAGCTCT-3’ and reverse- 5’-TCTGGTTGACCATCAAATATTCC-3’ and the forward primer was used for sequencing to identify those with monoallelic mutations predicted to result in premature termination of translation.

### RNA sequencing of isogenic *BRCA2* haploinsufficient vs. wild-type T-ALL cells

RNA was extracted from BRCA2 wild-type or heterozygous mutant clones using the RNeasy Mini Kit (Qiagen) according to the manufacturer’s instructions. RNA samples were then treated with Ambion Turbo DNAse (Thermo Fisher Scientific, Waltham, MA), and DNA contamination was confirmed to be <10% for all samples. RNA quantity was determined using the Qubit RNA Assay Kit (Thermo Fisher Scientific) and RNA quality was determined on an Agilent Bioanalyzer using the RNA Pico Kit (Agilent, Santa Clara, CA). Using the NEBNext Ultra RNA Library Prep Kit for Illumina (New England Biolabs, Ipswich, MA), 50–100 ng of total RNA was converted into a DNA library following the manufacturer’s protocol. Following library construction, DNA libraries were assessed for quality control. Library quantity was determined using the Qubit High Sensitivity DNA Kit (Thermo Fisher Scientific) and library size was determined using the Bioanalyzer High Sensitivity Chip Kit (Agilent). Finally, libraries were put through qPCR using the Universal Library Quantification Kit for Illumina (Kapa Biosystems, Wilmington, MA) and run on the 7900HT Fast qPCR machine (Applied Biosystems, Foster City, CA). Libraries passing quality control were diluted to 2 nM using sterile water and then sequenced on the NextSeq500 (Illumina, San Diego, CA) at a final concentration of 2 pM on a paired end flowcell with 75 sequencing cycles in each direction, following all manufacturer protocols. Alignments were performed with STAR aligner (version 2.3.1z4) against the hg19 w ERCC92 genome (ftp://ftp.ensembl.org/pub/release-75/fasta/homo_sapiens/dna/). Aligned files were processed to primary (raw) read counts using featureCounts, and primary read counts were normalized using DESeq2. Gene set enrichment analysis was performed using GenePattern (http://software.broadinstitute.org/cancer/software/genepattern). RNA-seq data are available in NCBI GEO (https://www.ncbi.nlm.nih.gov/geo), accession number GSE126780.

### UV treatment in isogenic *BRCA2* haploinsufficient vs. wild-type T-ALL cells

*BRCA2* haploinsufficient Jurkat T-ALL clones W4 and W5, together with parental *BRCA2* wild-type cells transduced with Cas9 alone, were suspended in 100 μL and plated at 10 million cells/ml in a 6-well plate. Cells were exposed to UV-C using the Stratalinker 2400 (Stratagene, La Jolla, CA) at 30 J/m^2^ or no UV. Cells were then plated at 0.1 million cells/mL in a 96-well plate, and cell viability was measured by CellTiter-Glo Cell Viability Assay after 96 hours.

### In vitro drug treatment of isogenic *BRCA2* haploinsufficient vs. wild-type T-ALL cells

*BRCA2* haploinsufficient Jurkat T-ALL cells or their Cas9-transduced *BRCA2* wild-type controls were plated at 0.1 million cells/ml in 96 well plates, and treated with vehicle (DMSO or PBS) control and at the indicated concentrations of VE-821 (SelleckChem, S8007), Topotecan (SelleckChem, S1231), KU60019 (SelleckChem, S1570), Mitomycin C (Sigma, M4287), PF-477736 (SelleckChem, S2904), Olaparib (SelleckChem, S1060), CCT-241533 (Tocris, 4968), Etoposide (Sigma, E1383), C527 (ApexBio, A8693), PHA-767491 (SelleckChem, S2742), MK-1775 (SelleckChem, S1525) for 96 hours. Cell viability was measured by CellTiter-Glo Luminescent Cell Viability Assay (Promega, Madison, WI).

For 15-day treatment of BRCA2-haploinsufficient vs. wild-type controls, clone W4, clone W5, and parental Cas9 control cells were plated at 0.1 million cells/ml and split every 4 days at 1:9 in growth media (RPMI1640 with 10% FBS) in the presence of vehicle or drug. Cells were treated with VE821 (1 μM), AZD6738 (AstraZeneca, 0.25 μM), or vehicle (DMSO), and cell viability was measured by CellTiter-Glo Luminescent Cell Viability Assay when cells were split. Cell counts of drug-treated cells are normalized to those in vehicle-treated cells at that time point.

### Mice

NOD rag gamma (NRG) mice were purchased from Jackson Laboratory (Bar Harbor, ME; strain #007799). 6 to 8-weeks old male NRG mice were used for experiments, and mice were randomly assigned to experimental groups. Mice were handled in accordance with Good Animal Practice as defined by the Office of Laboratory Animal Welfare, under approval from the Boston Children’s Hospital Institutional Animal Care and Use Committee (protocol # 18-09-3784R).

### In vivo drug treatment of isogenic *BRCA2* haploinsufficient vs. wild-type T-ALL cells

For in vivo experiments, *BRCA2* haploinsufficient and their wild-type Cas9-expressing controls were transduced with either EGFP or dTomato expressing constructs. EGFP or dTomato protein-coding sequences were amplified in an attB-flanked PCR product from EX-EGFP-LV105 (Genecopoeia) or pUltra-Chili (https://www.addgene.org/48687/), respectively, and then Gateway-cloned into pLenti CMV Puro DEST (https://www.addgene.org/17452/). Lentivirus was generated as described above, and *BRCA2* haploinsufficient clone W5 cells and parental Cas9 control cells were transduced with either pLenti CMV Puro DEST EGFP or dTomato lentivirus, as indicated. EGFP or dTomato positive cells were sorted using a Facsaria II (BD Biosciences). Sorting efficiency (>99.5%) was confirmed by FACS analysis before starting experiments. Cells were then mixed at 1:1 ratio, and injected by tail vein injection (0.5 million cells/mouse) into NRG mice. Mice were treated with irradiation (450 cGy) 3 hours prior to transplantation. Four days post-injection, treatment began with either vehicle (40% propylene glycol, 50% sterile water, and 10% DMSO) or AZD6738 (25 mg/kg) in this same vehicle by oral gavage, twice daily, for 12 days. Mice were anesthetized by 2% isoflurane (v/v) prior to oral gavage. Mice were monitored daily and supportive care (hydrogel, baconbites, and 300 μl normal saline solution via subcutaneous injection) were administered daily for the duration of the experiment. Mice were euthanized using carbon dioxide at day 18 after leukemia injection, the bone marrow was isolated and dissociated through a 40 μM mesh filter, and red blood cells were lysed using Red Blood Cell Lysis reagent (BD Biosciences). EGFP and dTomato fluorescence were assessed by flow cytometry using the LSRFortessa cytometer (BD Biosciences). Data were analyzed using FlowJo version 10.4.2.

### Statistical analyses

Differences in categorical data were assessed via Fisher’s exact test. Differences in continuous data were assessed via the Welch t-test with Bonferroni correction for multiple hypothesis testing when variance was similar among the groups (standard deviations < 2-fold different), or via analysis of variance (ANOVA) with Dunnett’s adjustment for multiple hypothesis testing when variance was similar. Differences in event-free and overall survival were assessed by the log-rank test, and time-to-event distributions were estimated via the Kaplan-Meier method.

## Results

### Fanconi-BRCA mutations in human T-ALL

**Fig 1:**
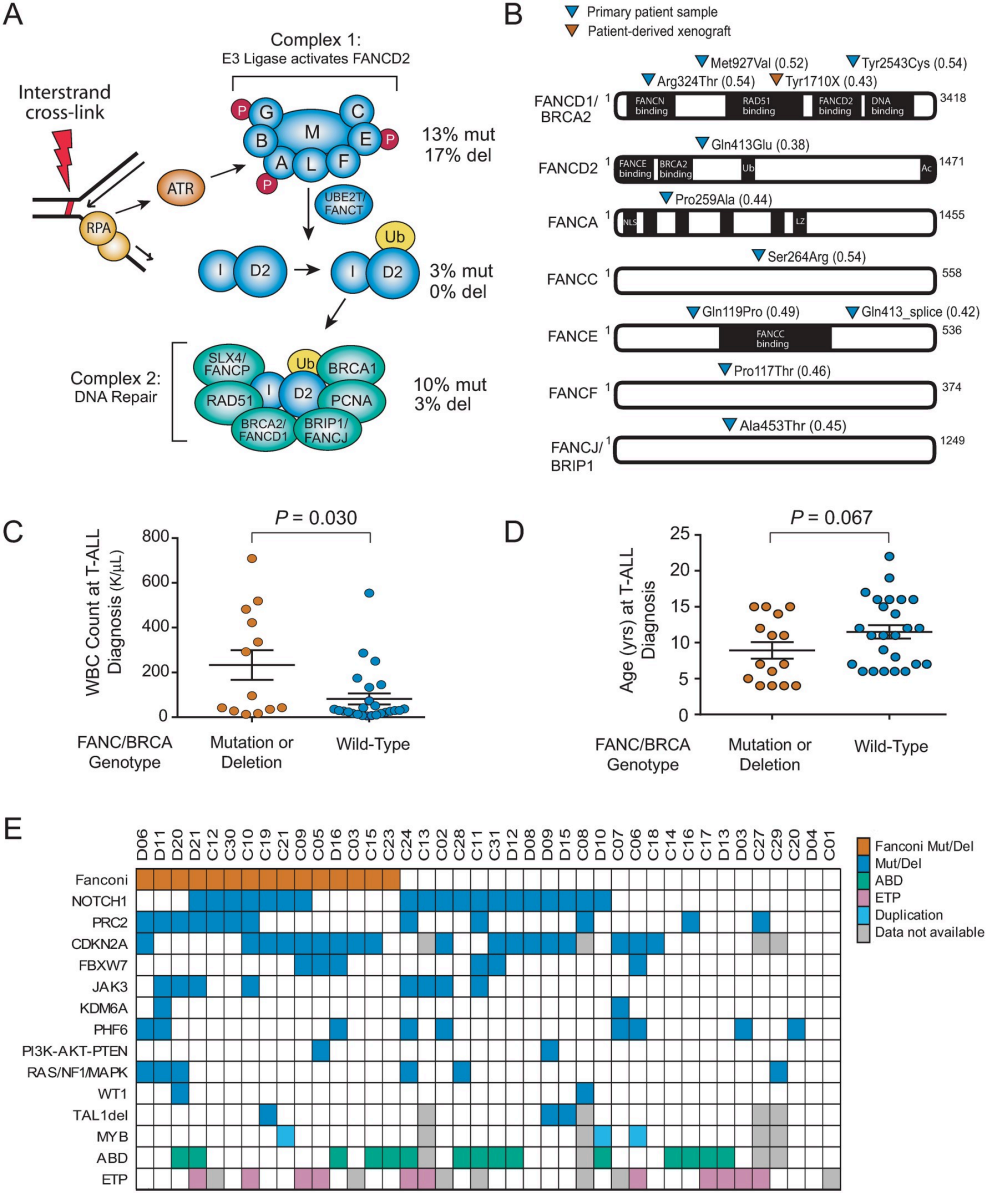
Fanconi-BRCA pathway mutations in childhood T-ALL. (A) Childhood T-ALL diagnostic specimens were analyzed by targeted exon sequencing and array CGH. The percentage of cases with mutations or deletions in each of the Fanconi complexes is shown on a simplified schema of the Fanconi pathway, which functions in the repair of DNA inter-strand crosslinks. (B) Mutations identified by targeted exon sequencing are shown for each of the mutated Fanconi-BRCA pathway genes. Allele fraction of the mutation is shown in parentheses. (C-D) Comparison of white blood cell (WBC) count (C) or patient age (D) at the time of T-ALL diagnosis in cases with or without Fanconi gene alterations. Significance assessed by two-sided Wilcoxon rank-sum test. (E) Co-occurrence of Fanconi gene mutations with additional T-ALL oncogenic lesions identified by sequencing and copy number analysis.

**Fig 2:**
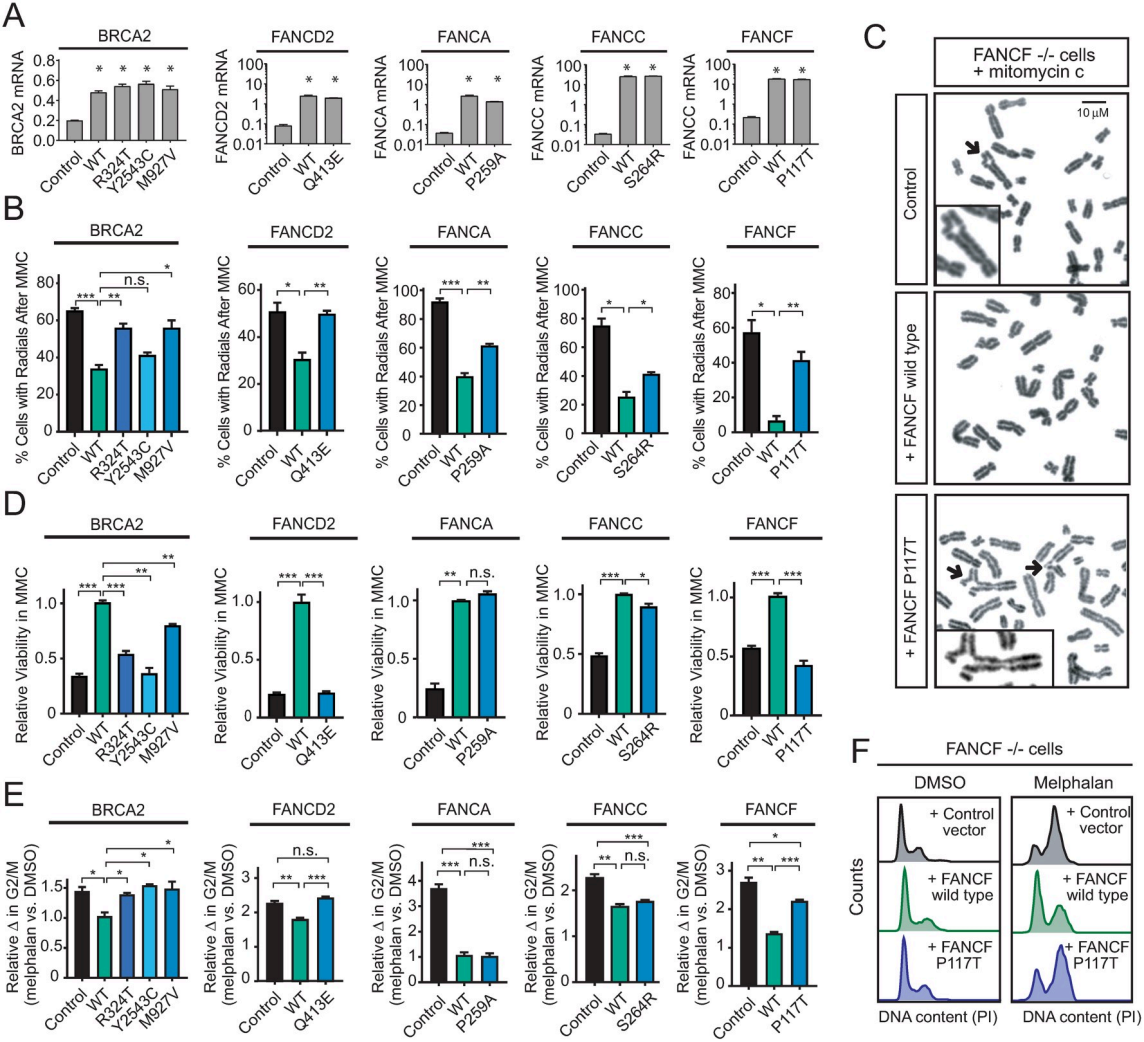
Fanconi-BRCA mutations in T-ALL encode pathogenic alleles. A panel of cell lines deficient for *BRCA2* (VU423), FANCD2 (PD20), FANCA (GM6914), FANCC (PD331) or FANCF (EUFA121.L) were transfected with pcDNA3 (BRCA2) or retrovirally infected with PMMP expression constructs encoding the wild-type (WT) gene (FANCD2, FANCA, FANCC, FANCF), control vector, or each of the indicated point mutant alleles identified in T-ALL patient samples. (A) Relative mRNA expression of the transduced genes was assessed by Q-RT-PCR analysis. All units are % of β-actin control. (B) The indicated cells were treated with mitomycin C (MMC; 20 ng/mL) for 48 hours, and radial chromosome formation was assessed by metaphase spread analysis. Radial chromosome formation was assessed in a minimum of 25 metaphases from each of three independent biologic replicates for each condition, and are plotted as mean +/- s.e.m. (C) Representative metaphase spread from the experiment shown in (B), with black arrows denoting radial chromosomes. (D) Relative viability was assessed by CellTiterGlo in the indicated cells transduced with the indicated constructs, following treatment with mitomycin C (MMC) at the following dosing: BRCA2 cells, 500 nM; FANCD2 cells, 100 nM; FANCA cells, 100 nM; FANCC cells, 500 nM; FANCF cells, 50 nM. (E) Cell cycle analysis was performed by PI staining in each of the transduced cell lines following treatment with melphalan at 0.25 μg/ml (BRCA2, FANCD2, FANCA, FANCC) or 0.5 μg/ml (FANCF) for 48 hours. The change in the percentage of cells in G2/M is shown relative to that in DMSO vehicle-treated cells. (F) Representative histograms of DNA content (PI) for each of the FANCF transduced cells in (E) are shown. All bar charts represent the mean +/- s.e.m. of three independent biologic replicates, with significance assessed by Welch t-test with Bonferroni adjustment for multiple comparisons as appropriate. ***, *P* ≤ 0.001; **, *P* ≤ 0.01; *, *P* ≤ 0.05; n.s., *P* > 0.05.

To further unravel the genetic basis of T-ALL, we applied targeted exon sequencing and array CGH analysis to T-ALL diagnostic specimens collected from a cohort of children treated on contemporary clinical trials (S2 Table). Sequencing analysis revealed 10 missense or splice site alterations of Fanconi genes in 23% (n = 9 of 40) of T-ALL cases analyzed in our primary cohort (Figs 1A and 2B, S2 and S3 Tables). All mutations were present at variant allele fractions of 0.38–0.54, consistent with heterozygous clonal mutations in the bulk tumor population (S3 Table). All mutations were validated by Sanger sequencing (S1 Fig). To validate these findings in an independent cohort, we subjected an additional cohort of 69 independent primary T-ALL patient samples to targeted sequencing analysis. This revealed Fanconi-BRCA pathway mutations in 17% (n = 12 of 69) of these cases (S4 and S5 Tables), an incidence not significantly different than that in our original cohort (P = 0.6).

Sequencing of remission specimens of samples in the 8 Fanconi-BRCA mutant cases in our primary cohort in which induction chemotherapy successfully cleared the bone marrow of leukemia revealed that 6 of these mutations were somatic, whereas 2 were present in the remission specimen (S1 Fig), suggesting a germline origin. In addition, array CGH analysis of our primary T-ALL cohort revealed 8 deletions involving Fanconi genes as part of large multi-gene chromosomal aberrations in 19% (n = 7 of 36) of cases with sufficient material for array CGH analysis (Fig 1A, S2 Fig, S2 Table). Overall, 15 (38%) of the 40 T-ALLs in the primary T-ALL cohort analyzed had Fanconi-BRCA gene alterations. These cases had a significantly increased WBC count (Fig 1C), and tended to be younger at the time of T-ALL diagnosis (Fig 1D). Strikingly, all 5 T-ALL cases diagnosed before 6 years of age had Fanconi-BRCA mutations (5/5 vs. 10/25 in older patients, *P* = 0.005 by Fisher’s exact test). There were no significant differences in clinical responses between Fanconi-BRCA mutant vs. wild-type cases (S3 Fig). Fanconi alterations frequently co-occurred with mutations of *NOTCH1*, *CDKN2A* and polycomb repressive complex 2 genes (Fig 1E, S3 Table), genes with well-established roles in T-ALL pathogenesis [41, 42], but Fanconi-BRCA mutations were not significantly associated with specific molecular subtypes of T-ALL. A comparison of variant allele fractions for these mutations revealed that Fanconi-BRCA mutations were most likely clonal (S2 and S3 Tables).

### Fanconi-BRCA mutations identified in T-ALL encode pathogenic alleles

To test whether the identified Fanconi-BRCA mutations are pathogenic, we leveraged complementation analysis in a panel of cell lines with biallelic loss-of-function mutations in *BRCA2*, *FANCD2*, *FANCA*, *FANCC* or *FANCF* [43, 44]. We note that none of the mutations we identified are known to be pathogenic, and most have not been previously reported in Fanconi or BRCA mutation databases (S2 Table). Fanconi-deficient cells were transduced with expression constructs encoding point mutant alleles identified, the respective wild-type gene, or a negative control. The wild-type and mutant constructs were expressed at similar levels based on Q-RT-PCR and Western blot analysis (Fig 2A and S5 Fig). Transduced cells were treated with the DNA cross-linking agent mitomycin C, and radial chromosome formation was assessed by metaphase spread analysis. This revealed that most of the Fanconi-BRCA mutant alleles tested were impaired in their ability to rescue radial chromosome formation when compared to the wild-type gene (Fig 2B and 2C). For example, the FANCD2 Q413E allele was indistinguishable from a negative control, whereas FANCC S264R was approximately 75% as efficient as wild-type at reducing radial chromosome formation. All mutant alleles except FANCA P259A were also impaired in their ability to rescue Fanconi-deficient cells from the cytotoxicity of mitomycin C (Fig 2D). We then tested whether these mutant alleles were impaired in their ability to rescue G2/M arrest in response to melphalan, an alternative assay for canonical Fanconi pathway function [45], and found that most of the mutant alleles tested were inferior to their corresponding wild-type genes in this assay as well (Fig 2E and 2F). Thus, while some alleles are only modestly impaired, these data indicate that Fanconi-BRCA mutations identified in childhood T-ALL encode hypomorphic or null alleles.

**Fig 3:**
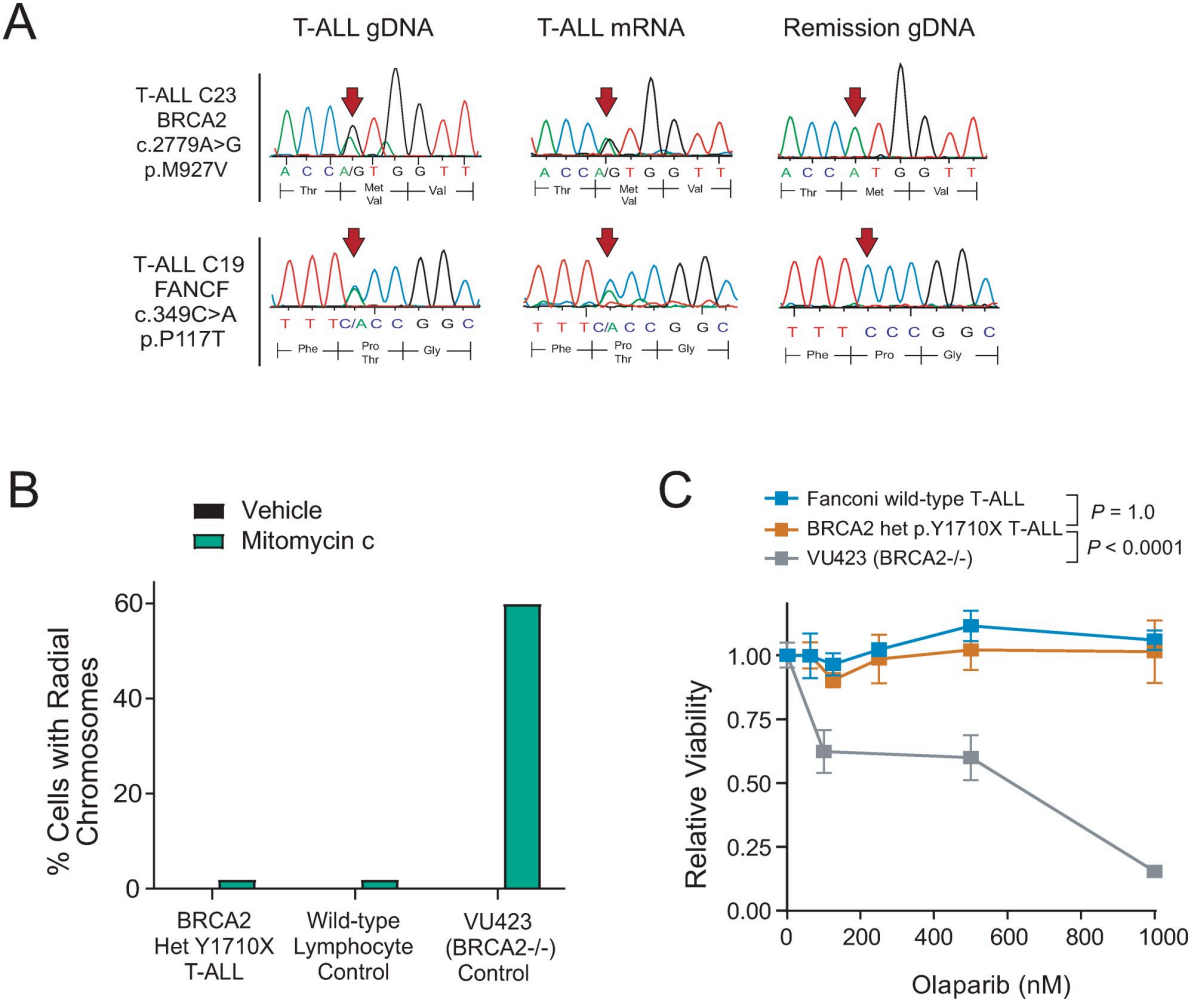
T-ALLs with monoallelic Fanconi-BRCA mutations retain expression of the wild-type allele. (A) Sanger sequencing of the mutant locus was performed on T-ALL diagnostic genomic DNA (gDNA), T-ALL mRNA, or remission gDNA collected from the indicated patients. Arrows denote the position of the mutation identified in T-ALL gDNA by next-generation sequencing. Sequencing from additional cases is shown in S1 Fig. (B) Cells from T-ALL PDX D115 harboring a heterozygous BRCA2 p.Y1710X truncating mutation, control Fanconi wild-type lymphocytes, or VU423 cells harboring biallelic *BRCA2* inactivation were treated with vehicle or 20 ng/ml of MMC for 48h, and radial chromosome formation was assessed by metaphase spread analysis. A minimum of 50 metaphase spreads per case were assessed. (C) The indicated cells were treated with the PARP inhibitor olaparib at the indicated doses for 5 days, and viability was assessed using CellTiter Glo. Each experiment shows the mean +/- s.e.m. of three biologic replicates. Significance was assessed at 1000 nM olaparib using a Welch t test with Bonferroni adjustment for multiple comparisons.

Inactivating the tumor suppressor activity of the Fanconi-BRCA pathway is generally thought to require biallelic gene mutations, and biallelic gene mutations are detectable in 90% of BRCA-mutant breast cancers [9]. However, there were no biallelic Fanconi-BRCA gene mutations identified in any T-ALL sample in our cohort (S2 Table). Moreover, Sanger sequencing from mRNA of Fanconi-mutant T-ALLs suggested that 8 of the 9 Fanconi-mutant cases retained mRNA expression of the wild-type allele (Fig 3A, S1 Fig). To test whether the wild-type allele is functional, we took advantage of the fact that cells with biallelic, but not monoallelic, Fanconi-BRCA pathway mutations have gross defects in the repair of DNA cross-links, detectable as hypersensitivity to inter-chromosomal fusions (radial chromosomes) in response to DNA cross-linking agents [46], and are hypersensitive to the cytotoxicity of PARP inhibitors [27, 47, 48]. Treatment of T-ALL patient-derived xenograft (PDX) cells harboring a heterozygous truncating *BRCA2* p.Y1710X mutation (Fig 3A) revealed that these cells lacked hypersensitivity to mitomycin C-induced radial chromosome formation (Fig 3B). These cells also harbored no hypersensitivity to the PARP inhibitor olaparib, at doses that were highly toxic to BRCA2-null VU423 cells (Fig 3C). While we lacked additional viable Fanconi-BRCA mutant T-ALL cells to confirm that these findings are generalizable to T-ALLs with upstream Fanconi gene mutations, these data suggest that T-ALLs are under little selective pressure to completely inactivate the wild-type allele.

### BRCA2 haploinsufficient T-ALL cells are hypersensitive to ultraviolet irradiation and ATR inhibition

**Fig 4:**
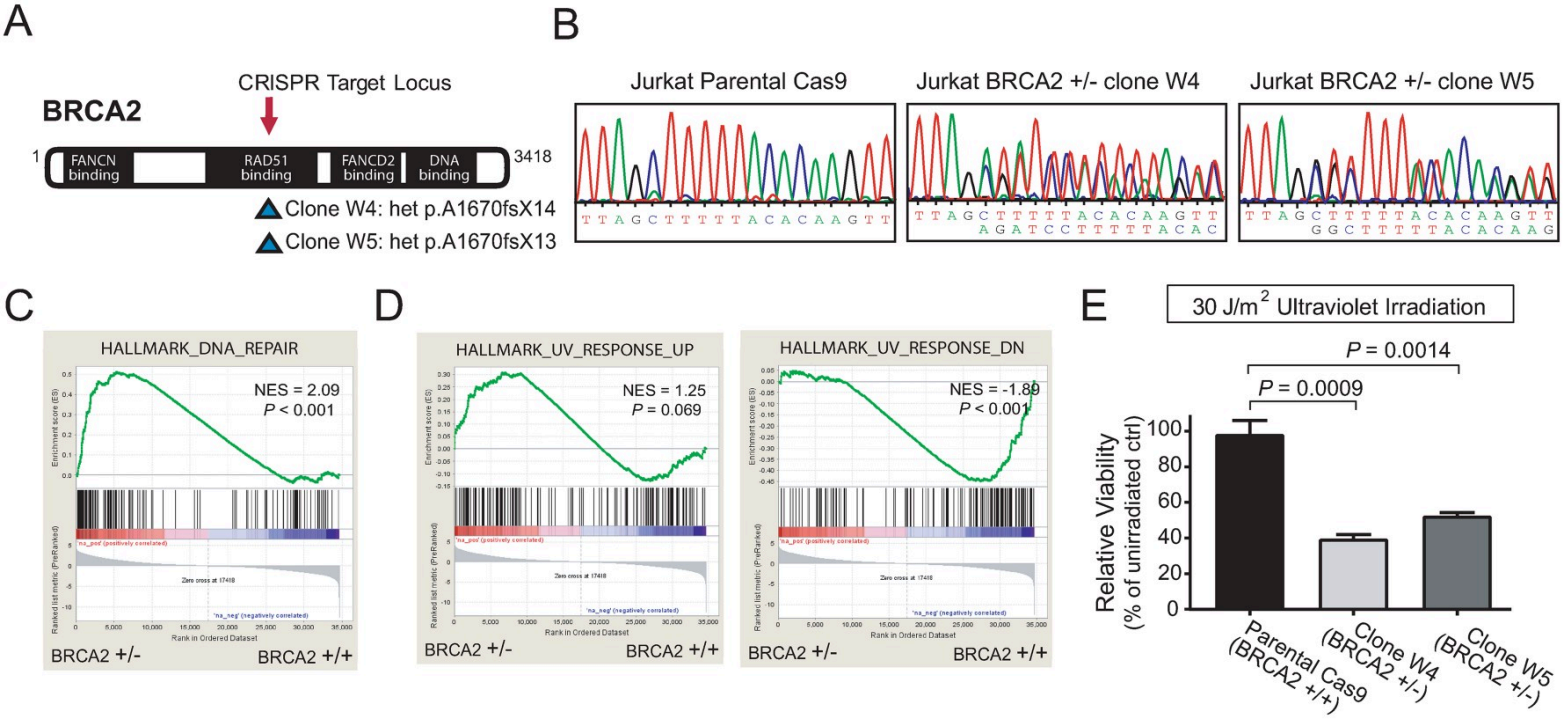
BRCA2 haploinsufficiency induces sensitivity to ultraviolet irradiation. (A-B) Cas9-expressing Jurkat T-ALL cells were transiently transduced with a guide RNA targeting the locus indicated in (A). Cells were single cell cloned, and Sanger sequencing identified the two clones with monoallelic frameshift mutations shown in (B). (C) RNA-seq was performed on the two *BRCA2* haploinsufficient clones, together with parental or Cas9-transduced Jurkat controls, and gene set enrichment analysis was performed. NES, normalized enrichment score. (D) An equal number of the indicated cells were subjected to 30 J/m2 ultraviolet irradiation, and viability was assessed at 96 hours. (E) Viability was normalized to that in non-irradiated controls. Significance was assessed by Welch t test.

We then turned our attention to the cellular consequences of *BRCA2* haploinsufficiency in T-ALL, because this was the single most commonly mutated Fanconi-BRCA gene in this cohort (Fig 1B). We first generated isogenic *BRCA2* wild-type versus haploinsufficient T-ALL cells in the human cell line Jurkat. We selected Jurkat cells because sequencing has identified no pathogenic mutations in *BRCA2* or other Fanconi-BRCA pathway genes [39]. Cas9-expressing Jurkat cells were transiently transduced with an integration-defective lentivirus encoding a guide RNA targeting exon 11 (the exon mutated in T-ALL PDX D115), and single-cell cloning revealed two independent clones with monoallelic mutations predicted to result in premature termination of translation (Fig 4A and 4B). There was no significant difference in relative viability of BRCA2 haploinsufficient versus parental clones (S6 Fig).

We began by performing RNA-seq analysis on these two *BRCA2-*haploinsufficient Jurkat clones, together with two *BRCA2* wild-type control clones (Cas9-expressing and untransduced parental Jurkat cells). Gene set enrichment analysis revealed that *BRCA2* haploinsufficiency was associated with increased expression of the Hallmark_DNA_Repair gene set (Fig 4C), representing genes whose coordinate expression is associated with DNA damage repair in a broad spectrum of experimental conditions [49]. *BRCA2* haploinsufficient cells were also characterized by increased expression of genes upregulated in response to ultraviolet (UV) irradiation, and decreased expression of genes downregulated in the UV response (Fig 4D), suggesting that *BRCA2* haploinsufficient cells might have limited reserves to tolerate UV irradiation. UV radiation induces pyrimidine dimers that are typically repaired by nucleotide excision repair or trans-lesion synthesis [50]. However, UV radiation during S phase does trigger activation of the Fanconi-BRCA pathway [51], and FANCD2 function is required for genomic integrity following UV-induced replication stress [52]. We found that *BRCA2-*haploinsufficient cells were selectively sensitized to UV irradiation at doses that had minimal toxicity to their isogenic wild-type counterparts (Fig 4E).

**Fig 5:**
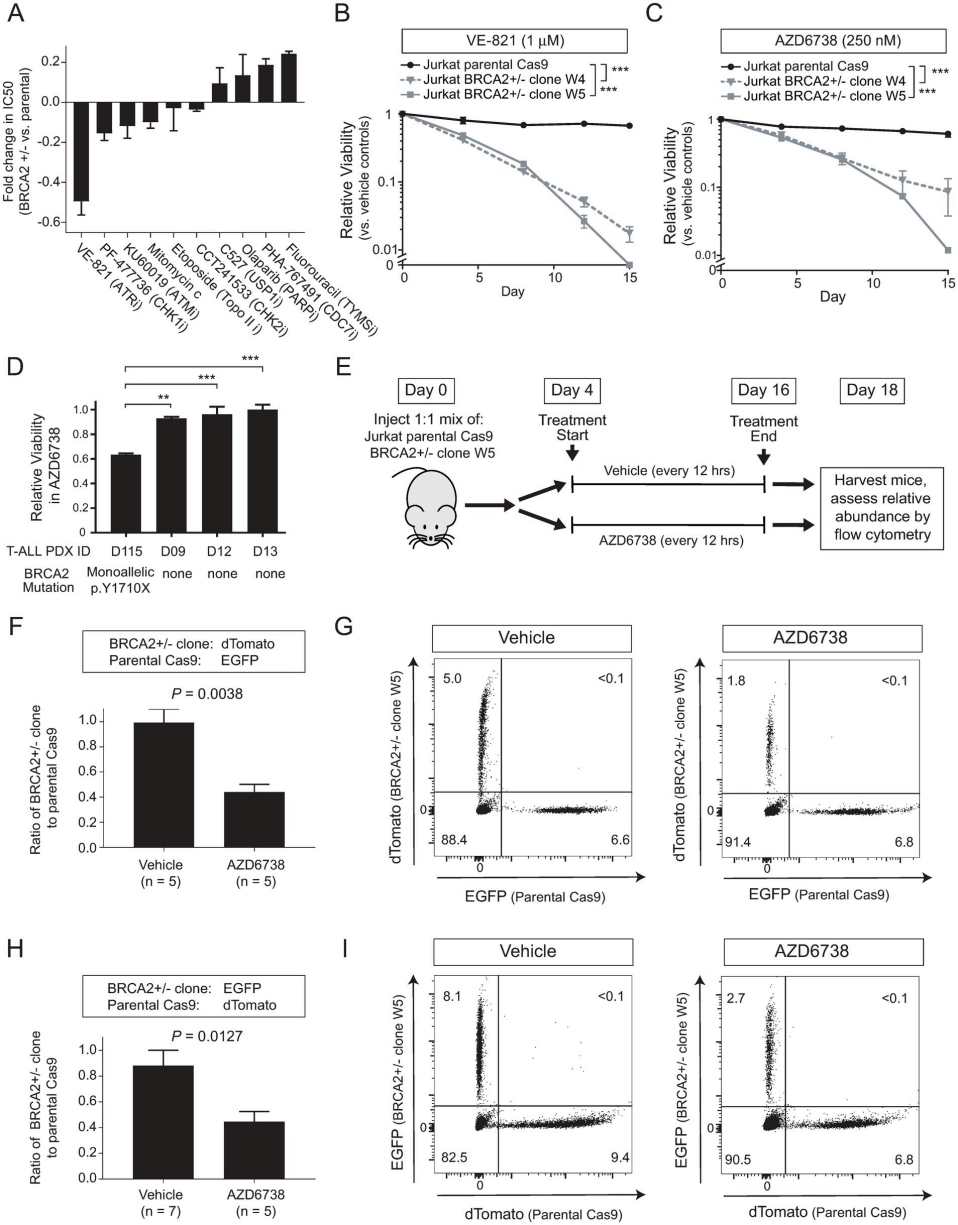
BRCA2 haploinsufficiency induces hypersensitivity to ATR inhibition. (A) Jurkat *BRCA2*-haploinsufficient clone W5 cells, and Cas9-transduced parental controls, were treated with the indicated drugs for 96 hours, and viability was assessed using CellTiter Glo. Graph denotes the mean +/- s.e.m. of the fold change in IC50 between BRCA2 +/- versus parental cells from triplicate experiments. Significance assessed by repeated measures two-way ANOVA. (B-C) The indicated BRCA2 haploinsufficient Jurkat clones, or Cas9-transduced parental controls, were treated with the ATR inhbitors VE-821 (1 μM) or AZD6738 (0.25 μM), and cell viability was assessed by CellTiter Glo at the indicated time points. Graphs denotes the mean +/- s.e.m. of biologic triplicates. (D) T-ALL patient-derived xenograft (PDX) cells were treated for 4 days with AZD6738 (0.8 mM), and viability was assessed by CellTiter Glo. Graph denotes the mean +/- s.e.m. of biologic triplicates. Significance assessed by one-way ANOVA with Dunnett’s correction for multiple hypothesis testing. (E) Schema of experimental design. Vehicle or 25 mg/kg/dose AZD6738 were administered by gavage every 12 hr. (F) Mice were injected with a 1:1 mix of dTomato-labeled *BRCA2* haploinsufficient clone W5 and EGFP-transduced parental Cas9 (*BRCA2* wild-type) cells, and treated as shown in (E). Abundance of each fluorescently labeled clone in the bone marrow was assessed by flow cytometry on day 18. Significance was assessed by two-sided Welch t-test. (G) Representative flow cytometry plots from the experiment shown in (E). (H-I) Mice were injected, treated and analyzed as in (F-G), with the fluorescent labels swapped: *BRCA2* haploinsufficient cells were transduced with EGFP, and parental Cas9 (*BRCA2* wild-type) cells were transduced with dTomato. ***, *P* ≤ 0.001; **, *P* ≤ 0.01; *, *P* ≤ 0.05; n.s., *P* > 0.05.

We then asked whether therapeutic vulnerabilities induced by *BRCA2* haploinsufficiency could be exploited using drugs. We screened a panel of small molecules selected based on their potential to trigger DNA replication-dependent toxicity, in *BRCA2* haploinsufficient clone W5 cells versus their isogenic wild-type counterparts (S7 Fig). This revealed that the ATR inhibitor VE-821 [53] was selectively toxic to cells harboring monoallelic *BRCA2* mutations following 4 days of treatment (Fig 5A). To confirm these findings, we then treated our two independent *BRCA2* haploinsufficient clones with either VE-821 or the structurally distinct ATR inhibitor AZD6738 [54], and found that ATR inhibition exhibits sustained differential toxicity to *BRCA2* haploinsufficient Jurkat T-ALL cells (Fig 5B and 5C). While T-ALL patient-derived xenografts are unable to be maintained in cell culture for more than a few days, short-term treatment of a panel of T-ALL PDX cells revealed that the ATR inhibitor AZD6738 was toxic only to those from a case harboring a monoallelic *BRCA2* truncating mutation (Fig 5D).

To test the in vivo therapeutic potential of ATR inhibition in *BRCA2* haploinsufficient T-ALL, we first transduced *BRCA2-*haploinsufficient clone W5 cells with EGFP, and their isogenic parental cells with dTomato. These cells were mixed in a 1:1 ratio, and injected into a cohort of immunodeficient mice. Four days after injection, mice were treated either with vehicle or the ATR inhibitor AZD6738 via gavage for 12 days, and relative fitness of *BRCA2* mutant versus wild-type clones was assessed by flow cytometry analysis of the bone marrow. This experimental design ensures that both clones are treated under identical conditions. *BRCA2* haploinsufficient versus wild-type clones had indistinguishable growth characteristics in vehicle-treated mice. However, we found that the ATR inhibitor AZD6738 was selectively toxic to T-ALL cells harboring a monoallelic *BRCA2* mutation (Fig 5F and 5G). To rule out the possibility that this result reflected differential toxicity of the EGFP versus dTomato fluorescent proteins, we repeated this experiment with the labels swapped, which revealed nearly identical results (Fig 5H and 5I). Thus, *BRCA2* haploinsufficiency selectively sensitizes T-ALLs to ATR inhibition.

## Discussion

The identification of a high frequency of pathogenic Fanconi-BRCA mutations implicates this pathway in the molecular pathogenesis of human T-ALL, a possibility first raised by the spontaneous development of thymic lymphomas in *Brca2-*deficient mice [17, 18]. While T-cell transformation can occur with biallelic Fanconi-BRCA pathway mutations [17–19], the fact that all mutations in human T-ALL appeared to be monoallelic suggests that partially impaired, rather than absent, Fanconi pathway activity may provide optimal fitness during T-cell transformation. This model posits that Fanconi-BRCA haploinsufficiency sufficiently impairs some functions of the pathway to collaborate with other oncogenic lesions during T-cell transformation. Haploinsufficiency for some downstream Fanconi-BRCA genes has previously been shown to induce replication stress and impair DNA repair [55, 56], and we found that *BRCA2* haploinsufficiency induced gene expression alterations suggesting activation of a DNA damage response, as well as hypersensitivity to UV radiation. However, the lack of selection for loss of the wild-type allele suggests that retaining some Fanconi pathway function is beneficial, presumably by suppressing the deleterious consequences of extensive genomic instability. The relative specificity of this observation to the T-cell lineage may reflect the hypersensitivity of normal T-cell progenitors to DNA damage-induced apoptosis [57]. However, this is not unique to the T-cell lineage, as *BRCA2* can function as a haploinsufficient tumor suppressor during pancreatic transformation [58].

It is worth noting that a recent whole-exome sequencing (WES) study of a large T-ALL cohort detected a significantly lower rate of Fanconi-BRCA mutations than that reported in our study [59]. We confirmed each of the mutations identified in our cohort by Sanger sequencing, which rules out the possibility of next-generation sequencing artifacts, and also validated our findings in an independent cohort. We believe this apparent discrepancy reflects the difficulty of distinguishing pathogenic mutations from non-pathogenic passengers by informatics alone. The authors of this recent WES study applied a strict algorithm for mutation calling, which required mutations to be previously known to be pathogenic or clearly predicted to disrupt protein structure [59]. This is entirely appropriate, because without this the list of mutations identified in genome-wide cancer sequencing studies would be dominated by non-pathogenic variants, which are much more common. However, an unavoidable consequence of this approach is a bias against calling mutations not previously known to be pathogenic. We note that most of the pathogenic Fanconi-BRCA mutations we identified were missense substitutions not previously known to be pathogenic, and their functional impairment was not well correlated with predicted pathogenicity by a range of existing informatics tools, in line with prior reports [60, 61]. We note that recent efforts using novel informatics algorithms designed to better predict pathogenic mutations have also revealed Fanconi-BRCA mutations in other tumor types in which these had not previously been recognized by large-scale sequencing efforts [62–64]. These findings highlight the complementary strengths of human cancer genomics and functional genetics to decipher the molecular pathogenesis of human cancer.

We found that haploinsufficiency for *BRCA2*, the most commonly mutated Fanconi gene in our cohort, induced a gene expression signature suggesting activation of a DNA damage response, and BRCA2 haploinsufficient cells were hypersensitive to UV radiation at doses that lacked detectable toxicity to their wild-type counterparts. These cells were also selectively sensitized to ATR inhibition, but not to drugs such as PARP inhibitors that are toxic only in the setting of biallelic (and not monoallelic) Fanconi-BRCA inactivating mutations [27, 47, 48]. Our data provide a rationale for the application of ATR inhibitors in T-ALLs with somatic BRCA gene mutations, and suggest the need to test this strategy in other tumors with monoallelic Fanconi-BRCA gene mutations. ATR inhibitors are currently in clinical trials for a broad spectrum of tumor types, and it will be of considerable interest to assess whether Fanconi-BRCA mutations provide a biomarker of response to ATR inhibition across human cancer subtypes.

## Supporting information

S1 Fig Confirmation of Fanconi-BRCA point mutations identified in childhood T-ALL by Sanger sequencing. Sanger sequencing was performed to confirm all Fanconi-BRCA point mutations identified in primary T-ALL patient samples in genomic DNA (left). Two additional cases are shown in Fig 3a. Sequencing of cDNA (middle) revealed that all samples but one (T-ALL C03) retained expression of the wild-type allele at the mRNA level. Sequencing of remission bone marrow or peripheral blood specimens in all cases that achieved a remission revealed that 6 of 8 mutations were somatic, whereas 2 mutations were present in the remission specimen. We lacked a non-hematopoietic germline sample to distinguish whether these mutations were germline or indicative of clonal hematopoiesis.
(PDF)

S2 Fig Fanconi deletions in childhood T-ALL. (A-D) Array CGH was performed on all T-ALL diagnostic specimens with sufficient material available, which revealed large heterozygous deletions involving FANCG (A), FANCC (B), SLX4 (C) and FANCA (D) in 8 (22%) of these 36 cases. The chromosome segment shown is indicated in the ideogram (left). Segmented array CGH copy number data is shown on the right, with each column representing an individual T-ALL patient sample. Color indicates the log2 copy number ratio, as indicated in the legend (bottom left).
(PDF)

S3 Fig Fanconi mutations are not associated with T-ALL treatment response. (A-B) Kaplan-Meier survival analysis of the 40 children with T-ALL in the primary cohort of cases in this study, from patients treated on clinical trials COG AALL0434 or DFCI 05001, comparing cases with a Fanconi gene mutation or deletion versus those without a Fanconi mutation identified (Fanconi wild-type). P values were calculated using the log-rank test. (C-D) Kaplan-Meier survival analysis from an independent validation cohort of 69 children with T-ALL treated on DFCI 05001. P values were calculated by log-rank test.
(PDF)

S4 Fig Western blot analysis of Fanconi-BRCA deficient cells transduced with wild-type or mutant expression constructs for complementation experiments shown in Fig 2. (A) FANCA-deficient cells GM6914 were transduced with empty vector, FANCA WT (WT) or FANCA P259A (P259A). (B) FANCC-deficient PD331 cells were transduced with empty vector, FANCC WT or FANCC S264R (S264R). (C) FANCF-deficient EUFA121 cells were transduced with empty vector (EV), FANCF WT (WT) or FANCF P117T (P117T). (D) FANCD2-deficient PD20 cells were transduced with empty vector (vector), FANCD2 WT (WT) or FANCD2 Q413E (Q413E). (E) BRCA2-deficient VU423 cells were transduced with Luciferase (Luc), BRCA2 WT (WT), BRCA2 Y2543C (Y2543C), BRCA2 R324T (R324T), and BRCA2 M927V (M927V) mutations. U2OS cells are shown as a positive control for BRCA2 expression.
(PDF)

S5 Fig The D115 T-ALL patient-derived xenograft harbors a BRCA2 heterozygous mutation. Sanger sequencing analysis of genomic DNA revealed the presence of a heterozygous *BRCA2* mutation resulting in premature termination of translation in this patient-derived xenograft.
(PDF)

S6 Fig Baseline viability of BRCA2 haploinsufficient vs. parental Cas9 isogenic clones. An equal number of cells were seeded at day 0, and cell growth was assessed at the indicated time points by CellTiter Glo analysis. Viability is shown relative to day 0.
(PDF)

S7 Fig Viability curves of BRCA2 haploinsufficient vs. parental Cas9 isogenic clones upon treatment with the drugs shown in Fig 5A. Cells were treated with the indicated drugs and doses, and cell viability was assessed by CellTiter Glo at 96 hours. Viability is normalized to that in vehicle-treated control for each cell type.
(PDF)

S1 Table Genes sequenced by targeted exon sequencing. (XLSX)

S2 Table Primary T-All patient samples analyzed in Primary Patient Cohort. (XLSX)

S3 Table Results of targeted exon sequencing in Primary Patient Cohort. (XLSX)

S4 Table Primary T-All patient samples analyzed in Validation Patient Cohort. (XLSX)

S5 Table Results of targeted exon sequencing in Validation Patient Cohort. (XLSX)

S6 Table Primers used for PCR amplification, Sanger sequencing, site-directed mutagenesis, and quantitative PCR. (XLSX)
