# Supplementary figures and images for: Fanconi-BRCA pathway mutations in childhood T-cell acute lymphoblastic leukemia
Source: PLoS One. 2019 Nov 13;14(11):e0221288. doi: 10.1371/journal.pone.0221288 (PMC6853288; doi:10.1371/journal.pone.0221288)

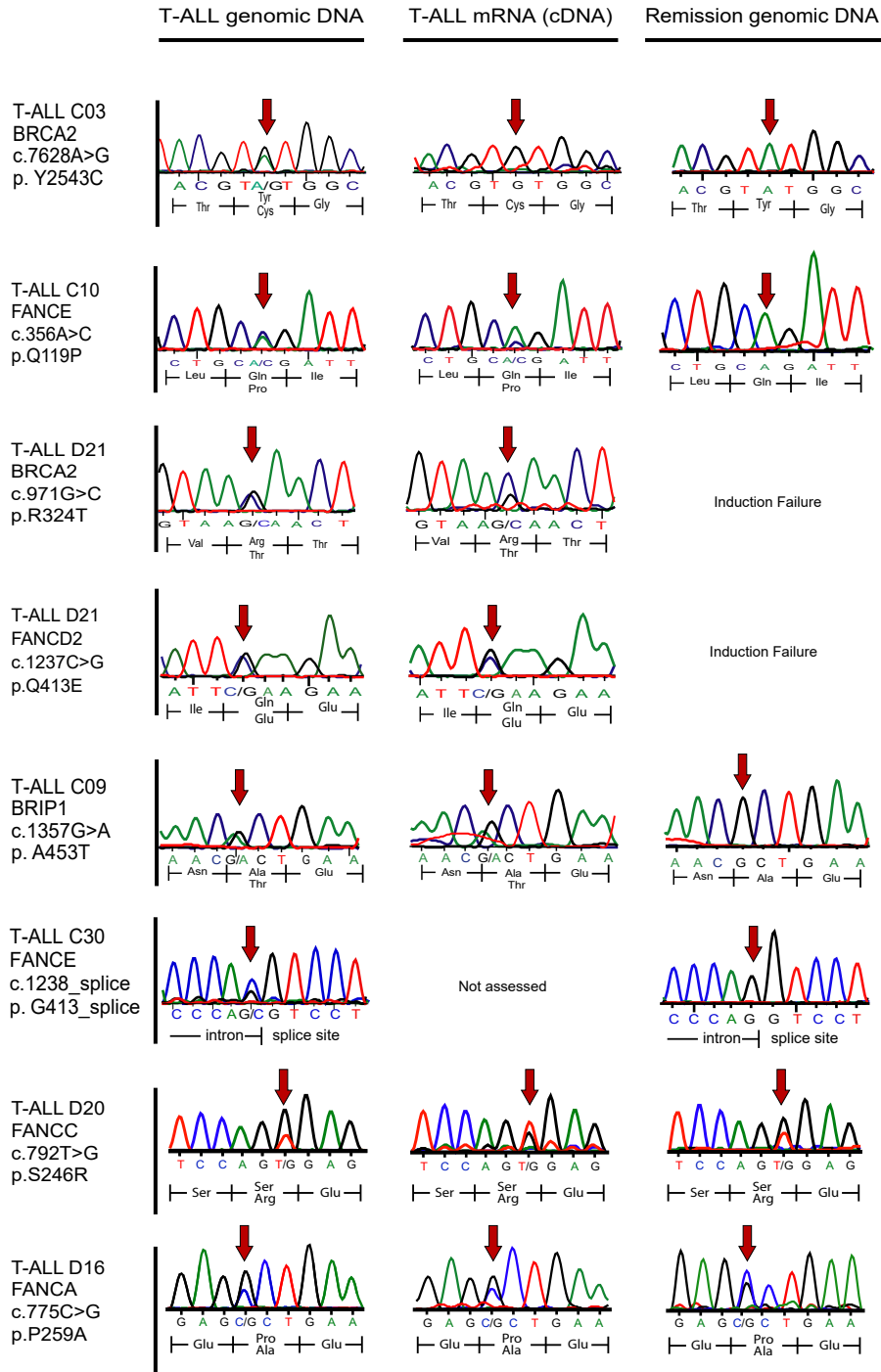

Supplement: S1 Fig — Sanger sequencing was performed to confirm all Fanconi-BRCA point mutations identified in primary T-ALL patient samples in genomic DNA (left). Two additional cases are shown in Fig 3a. Sequencing of cDNA (middle) revealed that all samples but one (T-ALL C03) retained expression of the wild-type allele at the mRNA level. Sequencing of remission bone marrow or peripheral blood specimens in all cases that achieved a remission revealed that 6 of 8 mutations were somatic, whereas 2 mutations were present in the remission specimen. We lacked a non-hematopoietic germline sample to distinguish whether these mutations were germline or indicative of clonal hematopoiesis. (PDF) [file pone.0221288.s001.pdf]

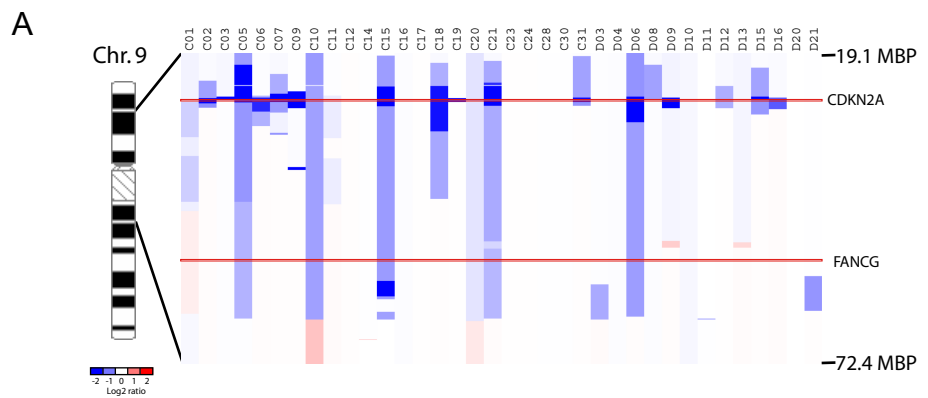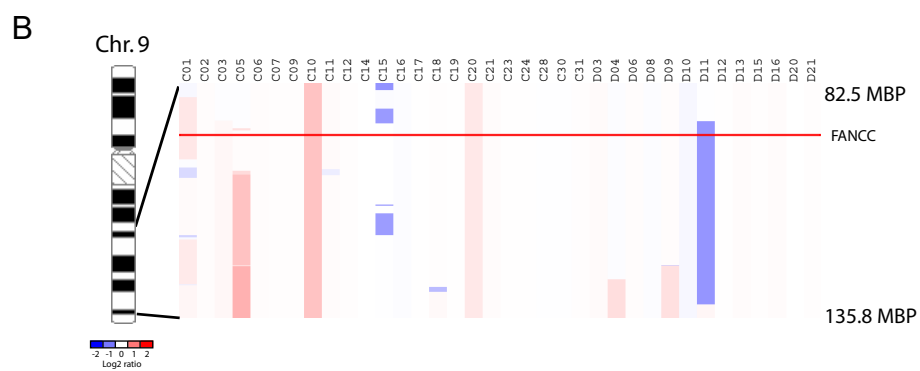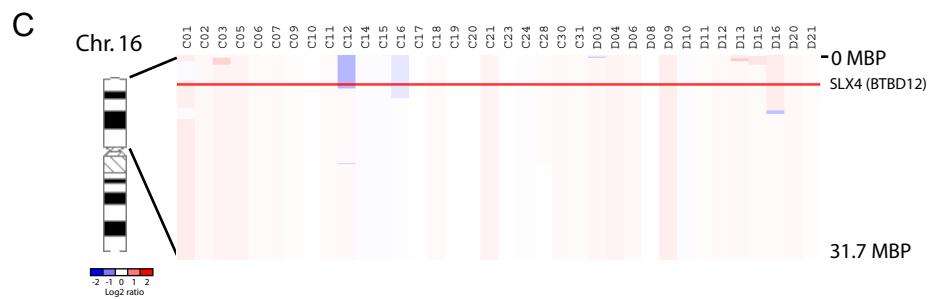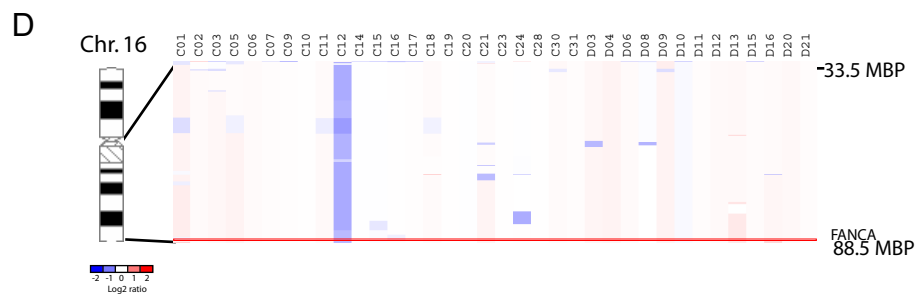

Supplement: S2 Fig — (A-D) Array CGH was performed on all T-ALL diagnostic specimens with sufficient material available, which revealed large heterozygous deletions involving FANCG (A), FANCC (B), SLX4 (C) and FANCA (D) in 8 (22%) of these 36 cases. The chromosome segment shown is indicated in the ideogram (left). Segmented array CGH copy number data is shown on the right, with each column representing an individual T-ALL patient sample. Color indicates the log2 copy number ratio, as indicated in the legend (bottom left). (PDF) [file pone.0221288.s002.pdf]

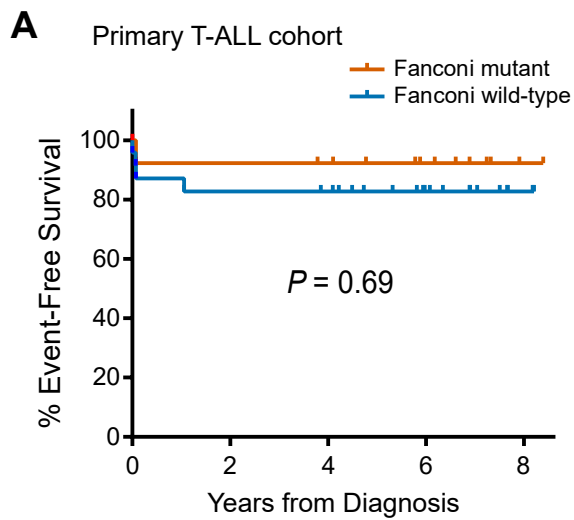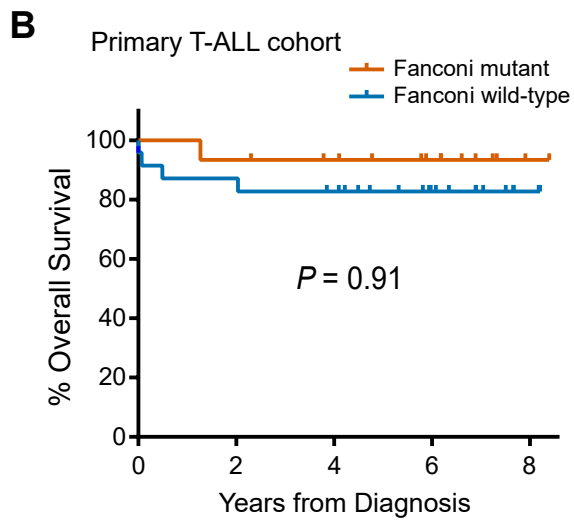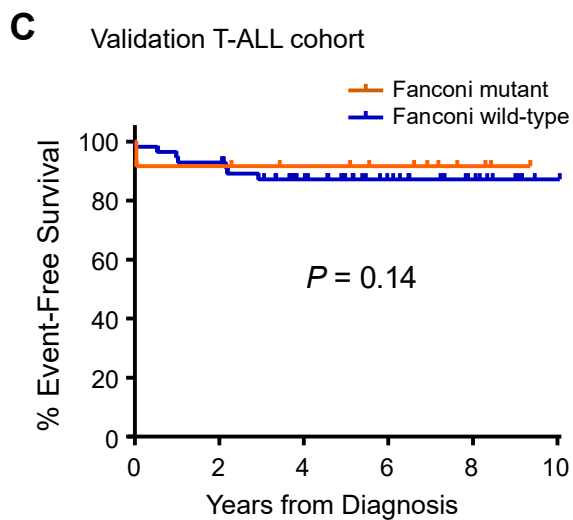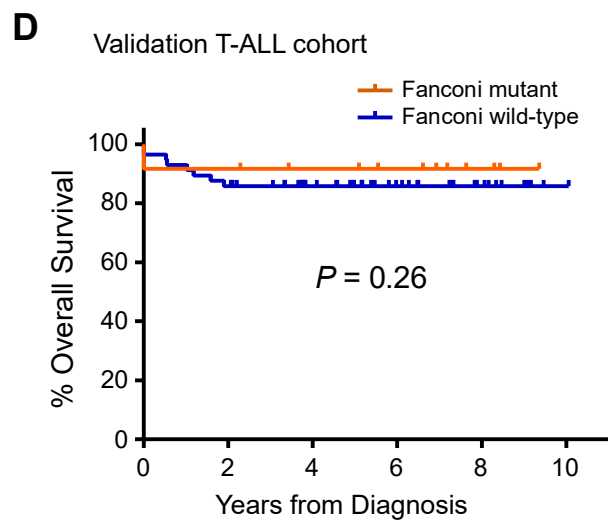

Supplement: S3 Fig — (A-B) Kaplan-Meier survival analysis of the 40 children with T-ALL in the primary cohort of cases in this study, from patients treated on clinical trials COG AALL0434 or DFCI 05001, comparing cases with a Fanconi gene mutation or deletion versus those without a Fanconi mutation identified (Fanconi wild-type). P values were calculated using the log-rank test. (C-D) Kaplan-Meier survival analysis from an independent validation cohort of 69 children with T-ALL treated on DFCI 05001. P values were calculated by log-rank test. (PDF) [file pone.0221288.s003.pdf]

## T-ALL Genomic DNA

PDX D115  
BRCA2  
c.5127\_5131TTATG>T  
p.Y1710X

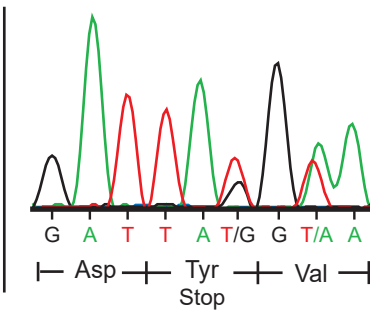

Supplement: S4 Fig — (A) FANCA-deficient cells GM6914 were transduced with empty vector, FANCA WT (WT) or FANCA P259A (P259A). (B) FANCC-deficient PD331 cells were transduced with empty vector, FANCC WT or FANCC S264R (S264R). (C) FANCF-deficient EUFA121 cells were transduced with empty vector (EV), FANCF WT (WT) or FANCF P117T (P117T). (D) FANCD2-deficient PD20 cells were transduced with empty vector (vector), FANCD2 WT (WT) or FANCD2 Q413E (Q413E). (E) BRCA2-deficient VU423 cells were transduced with Luciferase (Luc), BRCA2 WT (WT), BRCA2 Y2543C (Y2543C), BRCA2 R324T (R324T), and BRCA2 M927V (M927V) mutations. U2OS cells are shown as a positive control for BRCA2 expression. (PDF) [file pone.0221288.s004.pdf]

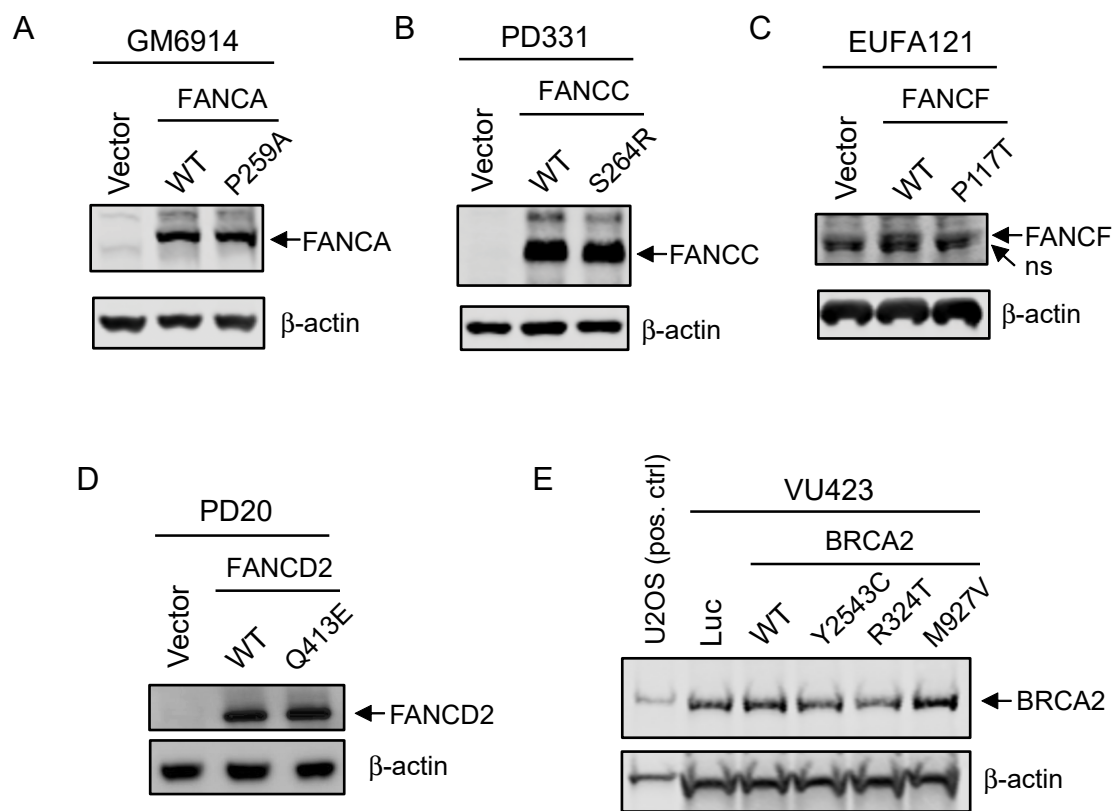

Supplement: S5 Fig — Sanger sequencing analysis of genomic DNA revealed the presence of a heterozygous BRCA2 mutation resulting in premature termination of translation in this patient-derived xenograft. (PDF) [file pone.0221288.s005.pdf]

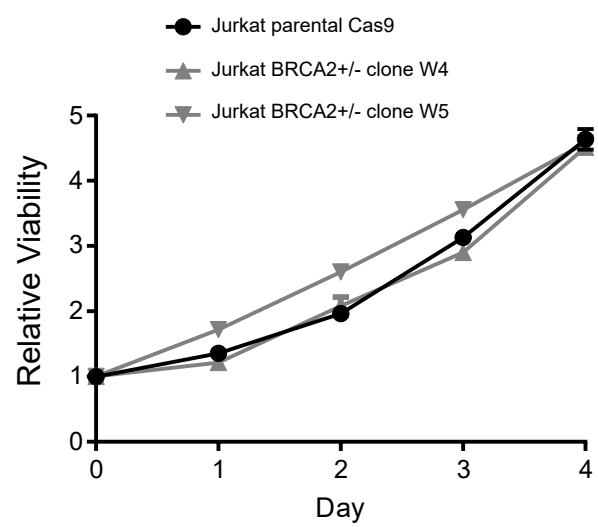

Supplement: S6 Fig — An equal number of cells were seeded at day 0, and cell growth was assessed at the indicated time points by CellTiter Glo analysis. Viability is shown relative to day 0. (PDF) [file pone.0221288.s006.pdf]

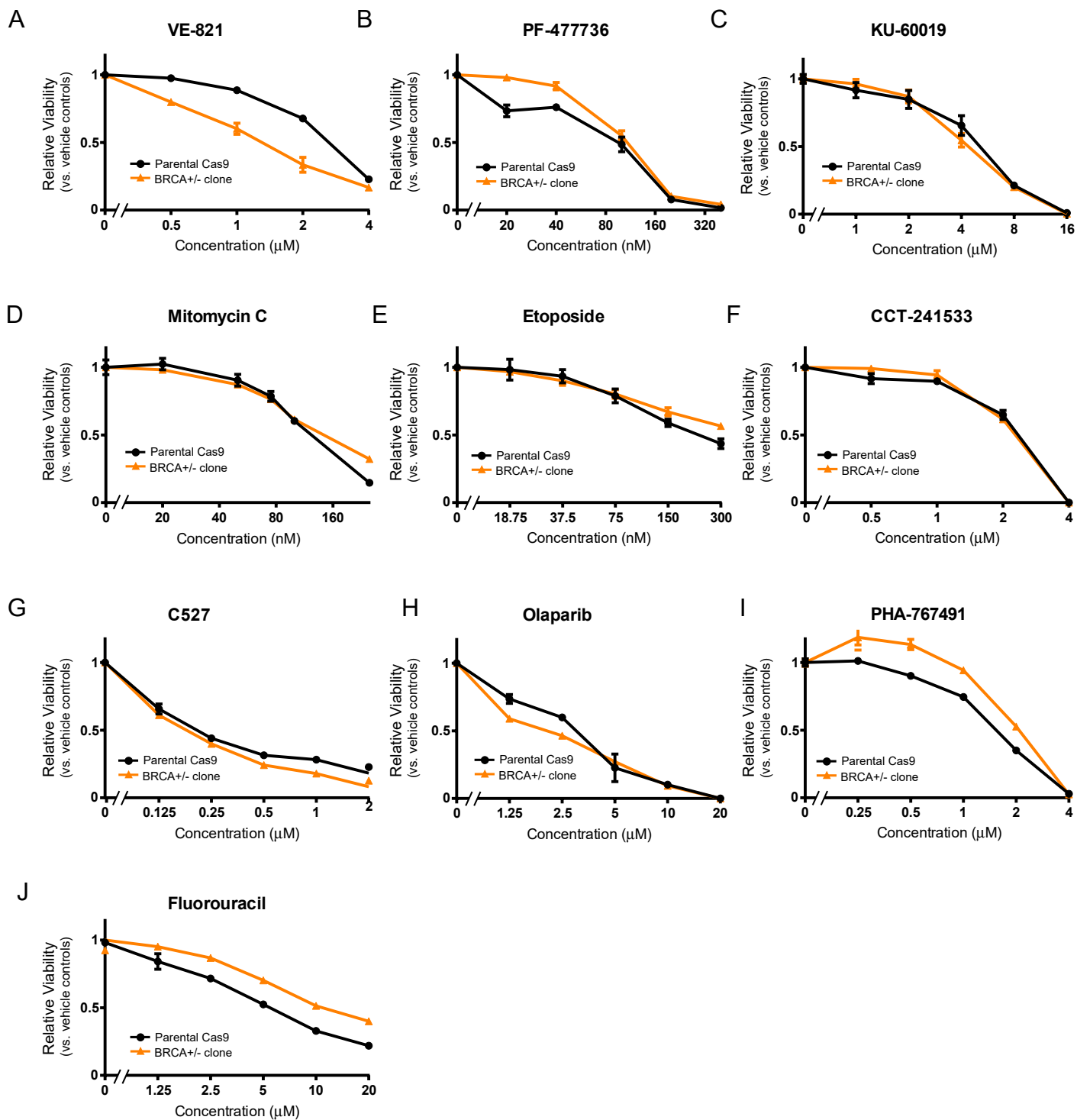

Supplement: S7 Fig — Cells were treated with the indicated drugs and doses, and cell viability was assessed by CellTiter Glo at 96 hours. Viability is normalized to that in vehicle-treated control for each cell type. (PDF) [file pone.0221288.s007.pdf]
